# Intrathoracic pressure regulation therapy applied to ventilated patients for treatment of compromised cerebral perfusion from brain injury

**DOI:** 10.1186/s13256-018-1720-1

**Published:** 2018-06-26

**Authors:** Anja K. Metzger, Nicolas Segal, Dai Wai Olson, Stephen A. Figueroa, Farid G. Sadaka, Catherine A. Krause, James R. Homuth, Nathaniel T. Burkhart, Robert T. Neumann, Keith G. Lurie, Victor A. Convertino

**Affiliations:** 1 University of Minnesota, Minneapolis, MN USA; 2 ZOLL Minneapolis, St. Paul, MN USA; 3 University of Texas Southwestern, Dallas, TX USA; 4 Mercy Hospital, St. Louis, MO USA; 5 University of Colorado, Denver, CO USA; 6 Battlefield Health & Trauma Center for Human Integrated Physiology, US Army Institute of Surgical Research, San Antonio, TX USA

**Keywords:** Blood gas analysis, Critical care, Critical illness, Hemodynamics/physiology, Humans, Intracranial hypertension, Intracranial pressure, Life support care/methods, Neurology, Nervous system diseases, Pressure, Traumatic brain injury

## Abstract

**Background:**

Reducing intrathoracic pressure in the setting of compromised cerebral perfusion due to acute brain injury has been associated with reduced intracranial pressure and enhanced cerebral perfusion pressure and blood flow in animals. Noninvasive active intrathoracic pressure regulation lowers intrathoracic pressure, increases preload, reduces the volume of venous blood and cerebral spinal fluid in the skull, and enhances cerebral blood flow. We examined the feasibility of active intrathoracic pressure regulation therapy in patients with brain injury. We hypothesized that active intrathoracic pressure regulation therapy would be associated with lowered intracranial pressure and increased cerebral perfusion pressure in these patients.

**Methods:**

At three institutions, active intrathoracic pressure regulation therapy (CirQlator™, ZOLL) was utilized for 2 consecutive hours in five mechanically ventilated patients with brain injury. A 30-minute interval was used to collect baseline data and determine persistence of effects after device use. End-tidal carbon dioxide was controlled by respiratory rate changes during device use. The intracranial pressure, mean arterial pressure, and cerebral perfusion pressure were recorded at 5-minute intervals throughout all three periods of the protocol. Results for each interval are reported as mean and standard deviation.

**Results:**

Intracranial pressure was decreased in all five patients by an average of 21% during (15 ± 4 mmHg) compared to before active intrathoracic pressure regulation (19 ± 4) (*p* = 0.005). This effect on intracranial pressure (15 ± 6) was still present in four of the five patients 30 minutes after therapy was discontinued (*p* = 0.89). As a result, cerebral perfusion pressure was 16% higher during (81 ± 10) compared to before active intrathoracic pressure regulation (70 ± 14) (*p* = 0.04) and this effect remained present 30 minutes after therapy was discontinued. No adverse events were reported.

**Conclusions:**

These data support the notion that active intrathoracic pressure regulation, in this limited evaluation, can successfully augment cerebral perfusion by lowering intracranial pressure and increasing mean arterial pressure in patients with mild brain injury. The measured effects were immediate on administration of the therapy and persisted to some degree after the therapy was terminated.

## Background

Brain injury alone is very common and affects three out of every 1000 Americans each year accounting for as many as 60,000 deaths and an estimated 70,000 to 90,000 patients with permanent neurological disabilities [1–3]. The economic burden of BI is immense. The direct and indirect costs of traumatic BI (TBI) in the USA have been estimated to be $48.3 billion annually. Survivor costs account for $31.7 billion and fatal brain injuries cost another $16.6 billion [4]. Accordingly, improving the care of patients with BI is a major health issue.

Several mechanisms can cause brain injury: TBI, subarachnoid hemorrhage (SAH), stroke, and intracerebral hemorrhage (ICH) are the most prevalent. Following brain injury, the contents of the intracranial compartment can be compressed due to increased intravascular volume (hyperemia) and tissue edema. Small increases in intracranial volume and the subsequent elevation in intracranial pressure (ICP) that impedes cerebral blood flow (CBF) can be accommodated by the movement of blood and cerebral spinal fluid (CSF) out of the cranium. However, as compensatory mechanisms become overloaded, cerebral circulation becomes compromised. A reduction in cerebral perfusion leads to ischemia, and, therefore, is a significant factor in secondary BI. Cerebral ischemia results in neuronal injury and cerebral edema. Cerebral circulation is generally assessed by measuring the cerebral perfusion pressure (CerPP), defined as the difference between the mean arterial blood pressure (MAP) and the ICP. It has been suggested that CerPP be maintained above 60–70 mmHg to minimize BI and optimize patient survival following brain injury [5, 6].

According to the Brain Trauma Foundation Guidelines, “Hypotension, occurring at any time from injury through the acute intensive care course, has been found to be a primary predictor of outcome from severe head injury for the health care delivery systems within which prognostic variables have been best studied. Hypotension is repeatedly found to be one of the five most powerful predictors of outcome and is generally the only one of these five that is amenable to therapeutic modification. A single recording of a hypotensive episode is generally associated with a doubling of mortality and a marked increase in morbidity from a given head injury” [7].

Reducing intrathoracic pressure (ITP) in the setting of acute brain injury has been previously associated with reduced ICP and enhanced CerPP and CBF in animal models [8–10]. Noninvasive active ITP regulation (aIPR) lowers ITP between positive pressure breaths, increases preload, reduces the volume of venous blood and CSF in the skull, and enhances arterial CBF. However, no translational studies have been performed in humans to confirm these results.

The purpose of this study was to evaluate the physiological response to application of aIPR in mechanically ventilated patients with compromised cerebral circulation. Adverse events were also evaluated as a measure of device safety. The hypothesis of this interventional study was that the physiological effects of aIPR therapy would improve cerebral circulation in patients with brain injury or intracranial pathology without causing any negative impact on other physiologic parameters.

## Methods

### Inclusion, exclusion, and discontinuation criteria

#### Inclusion criteria

To be included in the study, patients must: be ≥18 years of age; be intubated and able to be mechanically ventilated on a volume-controlled ventilation mode; be undergoing treatment for a head injury or other intracranial pathology; have a functioning ICP monitor at time of study; present with compromised cerebral perfusion per the attending physician for at least 30 minutes at any point within the past 24 hours prior to enrollment; have an arterial line in place or an alternative pressure measuring device (cuff) with continuous arterial pressure monitoring; have an oxygen saturation (SpO_2_) ≥ 90% with positive end-expiratory pressure (PEEP) requirement of no more than 7 cmH_2_O immediately prior to initiation of study aIPR treatment; be hemodynamically stable (defined as sustained MAP > 55 with or without the requirement for vasopressors; be in the intensive care unit (ICU) or about to undergo neurosurgery with planned placement of an invasive ICP monitor.

#### Exclusion criteria

Patients could not be included if they: had a cardiac or pulmonary injury impacting ITP and/or cardiac function; had radiologically evident pneumothorax or hemothorax; had neck injury resulting in neck swelling with jugular venous compression; had ongoing uncontrolled bleeding (including unsecured ruptured cerebral aneurismal hemorrhage or intraparenchymal expanding hemorrhage); had respiratory disease, for example, chronic obstructive pulmonary disease (COPD), interstitial lung disease, or other parenchymal or pulmonary vascular disease; had marked hypertension, which was defined as systolic blood pressure (SBP) > 180 mmHg; had heart failure; had a positive serum or urine pregnancy test or breast feeding for women.

#### Discontinuation criteria

aIPR treatment was discontinued if: the SBP was > 220 mmHg; the blood oxygen level dropped to < 85% with fraction of inspired oxygen (FiO_2_) delivered at 40% or < 90% with FiO_2_ at 100%; ICP increased by > 20% from baseline, resulting in an ICP > 20 that did not resolve within 5 minutes (baseline defined as first ICP measured during the 30-minute interval pre aIPR therapy), in the absence of a concomitant increase in CerPP.

### Prior or concurrent therapies to improve cerebral circulation

Patients who had been treated with other standard therapies to improve cerebral circulation during the index hospitalization could be included (for example, patients with poor cerebral circulation that persisted following mannitol therapy). It was desired that the aIPR be the only treatment during the study. However, at the attending physician’s discretion, patients who were receiving mannitol therapy and patients with pentobarbital coma were included as long as all patient selection criteria were met.

Patients were deeply sedated or under general anesthesia during aIPR treatment to prevent spontaneously triggered breathing or spontaneous breathing (not-triggered). Elimination of spontaneously triggered breathing was verified on the ventilator display before start of study and prior to collection of the study hemodynamic data. Neuromuscular blockade was used, as appropriate, at the physician’s discretion.

### Material

**Fig. 1:**
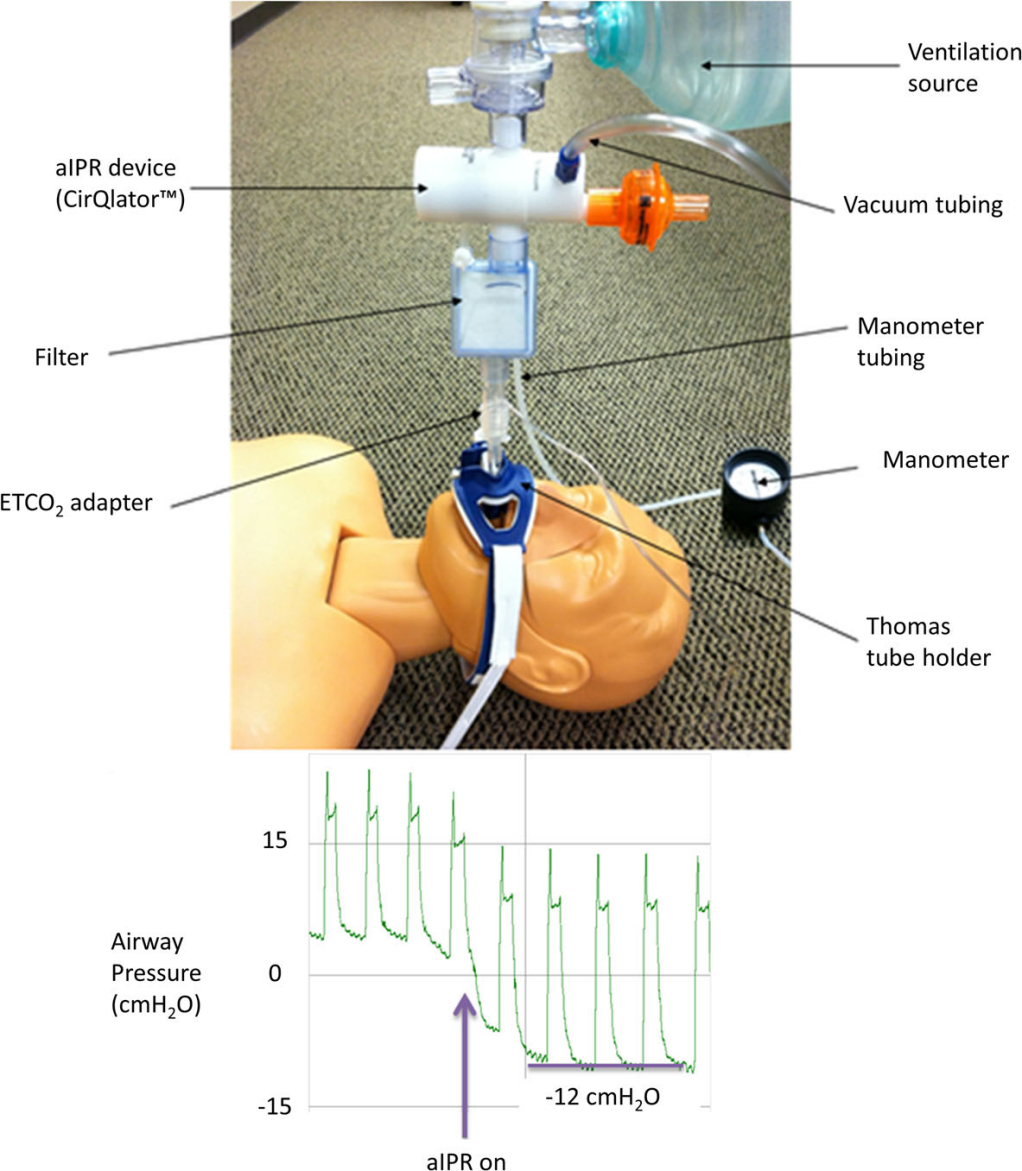
Active intrathoracic pressure regulation device (CirQlator™, ZOLL) used in the study to provide active intrathoracic pressure regulation therapy. The device is connected between a ventilation source and the patient’s airway circuit and the vacuum port tubing is connected to a regulated vacuum source. *aIPR* active intrathoracic pressure regulation, *ETCO*_*2*_ end-tidal carbon dioxide

Patients received aIPR treatment with the CirQlator™ (Fig. 1; ZOLL, Minneapolis, MN, USA). The advanced version of the CirQlator™ is called the CirQPOD™. This device recently received Section 510(k) clearance from the US Food & Drug Administration and allows the user to adjust the level of negative expiratory pressure between − 2 and − 12 cmH_2_O. The device utilized in this evaluation generates a negative airway pressure of − 12 cmH_2_O during the expiratory phase of ventilation between positive pressure breaths [11]. The device was connected to the patient’s airway circuit between the ventilator wye piece and the patient’s endotracheal tube. The device was also connected to an external vacuum source via tubing on a vacuum port. Airway pressure was measured with a re-useable manometer. During set-up, the vacuum source regulator was adjusted to provide the target vacuum level in the airway.

### Sites

Participants were enrolled at three hospitals: University of Texas Southwestern Medical Center (Dallas, TX), Mercy Hospital (St. Louis, MO), and University of Colorado Hospital (Aurora, CO).

### Measurement

Patient demographics, relevant clinical history, and type of neurosurgery or neuro-intervention including prior medication, sedation, and neuromuscular blockade, were recorded and summarized in Table 1. Continuous data capture capabilities were not available during the study so the following data were recorded every 5 minutes: blood pressure (BP), MAP, ICP, CerPP, respiratory rate (RR), SpO_2_, and end-tidal carbon dioxide (ETCO_2_). Three arterial blood gas (ABG) measurements, one draw 2–6 minutes prior to the end of each period (run-in, therapeutic, and run-out), were drawn to measure pH, partial pressure of carbon dioxide in arterial blood (PaCO_2_), partial pressure of oxygen in arterial blood (PaO_2_), bicarbonate (HCO^3−^), base excess (BE), and arterial oxygen saturation (SaO_2_). Adverse events were also recorded. Anticipated adverse events that were particularly monitored were device failure, sustained (> 30 seconds) relative increase in ICP by > 10 mmHg in the absence of a concurrent or greater rise in CerPP, elevation of absolute ICP to > 30 mmHg (assume ICP at baseline was less than 20 mmHg), sustained (> 30 seconds) decrease in MAP by > 20 mmHg, and reduction in SpO_2_ to < 90% for > 30 seconds.

**Table 1:** Patient demographics

		Patient 1	Patient 2	Patient 3	Patient 4	Patient 5
Age (years)		25	31	64	50	46
Gender		M	M	M	F	F
Height (cm)		180	173	188	170	163
Weight (kg)		85	65	84	103	95
Diagnosis		TBI (SDH with midline shift)	Cerebral edema	ICH	TBI	SAH
Side		Right	Both	Not reported	Left	Right
Delay between injury and aIPR (days)		3	3	5	1	16
Type of monitoring		Bolt	Bolt	EVD	Ventriculostomy	Camino
Sedation		Dexmedetomidine, fentanyl, propofol	Fentanyl, midazolam	Propofol	Unknown	Fentanyl, midazolam
Neuromuscular blockade		Cisatracurium	none	none	none	Cisatracurium
ICP (mmHg)	Before aIPR	16	19	17	16	25
	During aIPR	10	16	13	14	20
MAP (mmHg)	Before aIPR	80	78	66	96	112
	During aIPR	81	85	96	100	113
CerPP (mmHg)	Before aIPR	64	59	61	80	87
	During aIPR	71	69	85	86	93

*aIPR* active intrathoracic pressure regulation, *CerPP* cerebral perfusion pressure, *EVD* external ventricular derivation, *F* female, *ICH* intracerebral hemorrhage, *ICP* intracranial pressure, *M* male, *MAP* mean arterial pressure, *SAH* subarachnoid hemorrhage, *SDH* subdural hemorrhage, *TBI* traumatic brain injury

### Endpoints

The primary study endpoints included measured ICP and MAP with CerPP calculated at the end of each period. Secondary endpoints included times to maximum decrease of ICP and increase of CerPP during each aIPR treatment interval, change in CerPP 30 minutes after removal of the aIPR, and any other changes to hemodynamic or ABG values during aIPR treatment compared to baseline and following removal of aIPR.

### Protocol

**Fig. 2:**
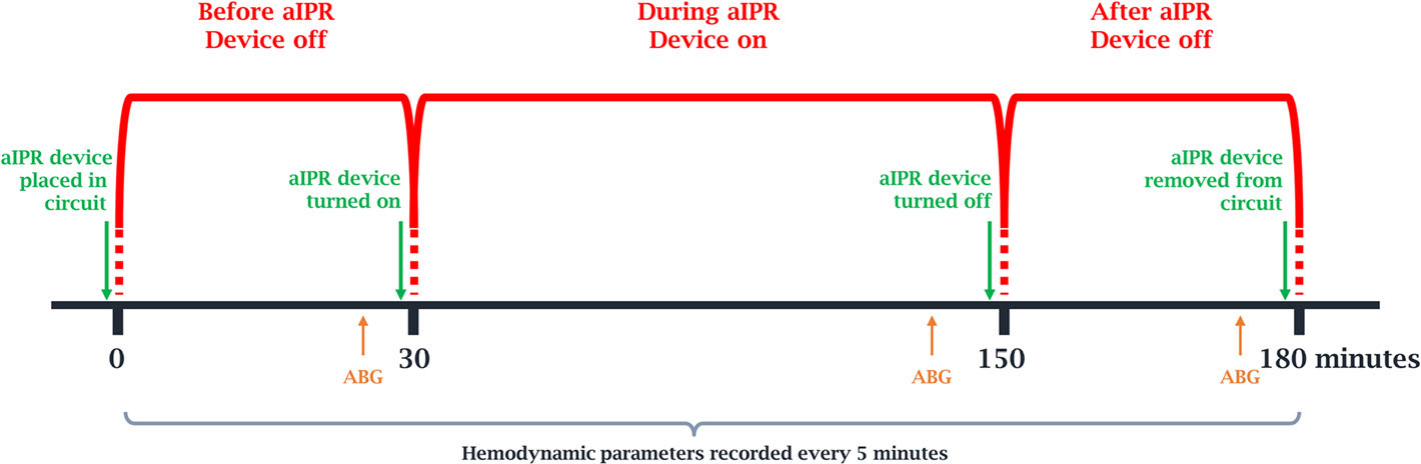
Study timeline. *ABG* arterial blood gas analysis, *aIPR* active intrathoracic pressure regulation

The timeline of the study is described in Fig. 2. Two 30-minute intervals pre aIPR and post aIPR were included in this study.

During the first period, “Before aIPR use” (30 minutes), the aIPR device was set in the patient’s airway circuit but not activated (vacuum source was not engaged), tidal volume (V_T_) was adjusted to 6–8 ml/kg plus ~ 50 ml to account for the dead space of the device in circuit, RR was adjusted to maintain normocarbia (ETCO_2_ of 35 ± 1 mmHg), and PEEP was set to 0 cmH_2_O.

During the second period, “During aIPR use” (120 minutes), the aIPR device was activated and the vacuum source was adjusted to ensure an airway pressure of − 12 cmH_2_O. Furthermore, RR was adjusted to maintain normocarbia.

During the third and last period, “After aIPR use” (30 minutes), the aIPR device was inactivated but was not removed from the airway circuit. RR was adjusted to maintain normocarbia.

### Statistical analysis

The primary study endpoint was CerPP calculated at the end of each period. Other measurements collected and analyzed included BP, MAP, ICP, CerPP, RR, SpO_2_, ETCO_2_, and ABG values. Statistical analyses were performed with two-tailed paired *t*-tests with SPSS 21.0 (IBM Corporation, USA). A *p*-value of < 0.05 was used to determine that any differences between measurements were not the result of chance alone. The *p*-values used to compare hemodynamic values are nominal and unadjusted. Values are expressed as means ± standard deviation (SD).

## Results

Five participants, with a mean age of 43 ± 15 years, were included in the study, two participants were female. The mechanisms of BI for participants enrolled were TBI (two participants), SAH (one participant), ICH (one participant), and undisclosed brain injury (one participant). Prior to aIPR therapy, three participants received treatment with mannitol. None of the participants had received pentobarbital. ICP monitoring was conducted by intracranial bolt in two patients, external ventricular drain in one patient, ventriculostomy in one patient, and a Camino in one patient. Two patients were receiving neuromuscular blockade.

**Fig. 3:**
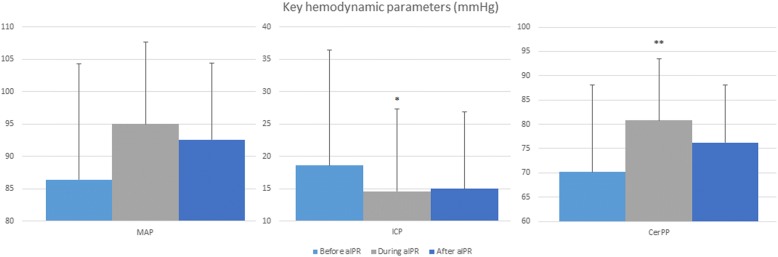
Key hemodynamic parameters at the end of each period (mean ± standard deviation, mmHg) before, during, and after active intrathoracic pressure regulation use. *aIPR* active intrathoracic pressure regulation, *CerPP* cerebral perfusion pressure, *ICP* intracranial pressure, *MAP* mean arterial pressure, * *p* = 0.005 and ** *p* = 0.04 between “before active intrathoracic pressure regulation” versus “during active intrathoracic pressure regulation”

**Fig. 4:**
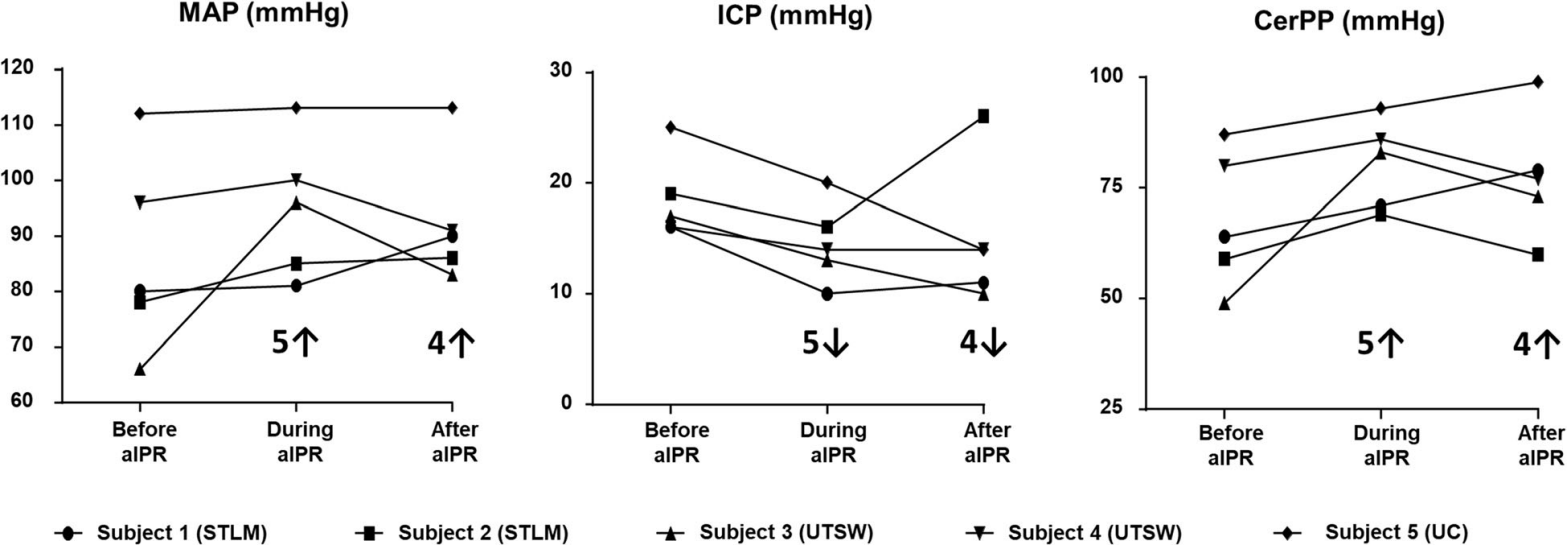
Key hemodynamic parameter changes for each participant. ↑ or ↓ indicates the number of patients whose values increased or decreased following each experimental condition. *aIPR* active intrathoracic pressure regulation, *CerPP* cerebral perfusion pressure, *ICP* intracranial pressure, *MAP* mean arterial pressure, *STLM* patients treated at Mercy Hospital (St. Louis, MO), *UC* a patient treated at University of Colorado Hospital (Aurora, CO), *UTSW* patients treated at University of Texas Southwestern Medical Center (Dallas, TX)

aIPR decreased ICP in all patients, resulting in an average ICP that was 21% lower (*p* = 0.005) during aIPR (15 ± 4 mmHg) compared to before aIPR (19 ± 4 mmHg) (Fig. 3 and Table 1). This effect on ICP was still present 30 minutes after aIPR was turned off as the ICP remained at 15 ± 6 mmHg, similar to the level observed at the end of aIPR therapy (*p* = 0.89). The effect was that CerPP was increased in all five patients, resulting in a 16% higher average value during aIPR (81 ± 10 mmHg) compared to before aIPR (70 ± 13 mmHg) (*p* = 0.04). This effect on CerPP was still present 30 minutes after the aIPR device was turned off (76 ± 14 mmHg) (*p* = 0.40 compared to aIPR). Even though aIPR was observed to cause an immediate effect once the device was activated, it took 35 minutes for that effect to be statistically different for ICP and 40 minutes for CerPP. The use of aIPR did not provide a statistical change in MAP. The average maximun effect on ICP was reached 62 ± 34 minutes after device activation and 49 ± 30 minutes after activation for CerPP. The effect on ICP and CerPP was maintained throughout the therapeutic interval. Figure 4 shows the effect of the device on each patient to demonstrate the effect according to the type of BI.

It is important to note that RR was adjusted to maintain normocarbia and no difference was found between intervals for ETCO_2_. No statistical differences were found for the other hemodynamic parameters shown in Table 2.

**Table 2:** Hemodynamic parameters before, during, and after active intrathoracic pressure regulation use

	Before aIPR	During aIPR	After aIPR
Systolic BP (mmHg)	129 ± 14	140 ± 21	138 ± 7
Diastolic BP (mmHg)	65 ± 16	72 ± 11	70 ± 10
HR (beats/minute)	84 ± 13	90 ± 17	80 ± 10
ETCO_2_ (mmHg)	33 ± 4	32 ± 5	32 ± 4
RR (breaths/minute)	27 ± 9	23 ± 5	25 ± 5
SpO_2_ (%)	97 ± 2	95 ± 2	96 ± 2

*aIPR* active intrathoracic pressure regulation, *BP* blood pressure, *ETCO*_*2*_ end-tidal carbon dioxide, *HR* heart rate, *RR* respiratory rate, *SpO*_*2*_ oxygen saturation

ABG analysis (Table 3) showed no effect of aIPR on pH, PaCO_2_, PaO_2_, HCO^3−^, or SaO_2_. However, BE during aIPR was improved.

**Table 3:** Arterial blood gas analysis before, during, and after active intrathoracic pressure regulation use

	Before aIPR	During aIPR	After aIPR
pH	7.4 ± 0	7.5 ± 0	7.4 ± 0
PaCO_2_	34.9 ± 6.1	31.2 ± 5.2	32.5 ± 5.7
PaO_2_	105 ± 29	104 ± 36	108 ± 36
HCO^3−^	22.1 ± 2.5	21.7 ± 2.2	21.4 ± 2.0
Base excess	−2.1 ± 2.5	−1.4 ± −2.8 *	−2.8 ± 2.4
SaO_2_	94.5 ± 3.5	95.5 ± 0.7	95.5 ± 2.1

*aIPR* active intrathoracic pressure regulation, *HCO*^*3−*^ bicarbonate, *PaCO*_*2*_ partial pressure of carbon dioxide in arterial blood, *PaO*_*2*_ partial pressure of oxygen in arterial blood, *SaO*_*2*_ arterial oxygen saturation, * *p* = 0.02 between “before active intrathoracic pressure regulation” versus “during active intrathoracic pressure regulation”

Three adverse events were reported during the study; however, no serious adverse events resulting in study interruption were reported. In one case, the participant had a SpO_2_ desaturation to 89%, however this was probably the result of a brief ventilator disconnect. In another case, the device made a strange noise when it was turned on. The device was immediately replaced with another device and patient care was not adversely affected. In the third case, the participant’s ICP increased by > 10 mmHg without a concurrent rise in CerPP, though this occurred at the end of the first pre-device interval of the protocol prior to the initiation of aIPR. Once aIPR was activated, the participant’s ICP was stabilized and ultimately decreased during the therapeutic period.

## Discussion

Current treatment of compromised cerebral circulation, typically manifested by a decrease in CerPP, includes: aggressive fluid resuscitation to maintain SBP greater than 90 mmHg; fluid as hypertonic saline and mannitol use to decrease ICP; control of PaO_2_ and PaCO_2_ through mechanical ventilation parameters to improve brain perfusion; sedation; lowering of the body temperature; prevention of jugular venous outflow obstruction by head elevation; pharmacological therapy; CSF drainage; and decompressive craniotomy. Treatments are applied in a step-wise fashion until satisfactory CerPP is achieved.

This translational study shows for the first time that 2 hours of continuous aIPR can decrease ICP and improve CerPP in patients with BI. This finding confirms what was previously shown in animal studies of brain injury and 10-minute applications of aIPR in patients with brain injury [8, 12]. In a swine model of brain injury, aIPR was demonstrated to significantly improve CerPP [8]. In the same study, this improvement in CerPP resulted in an improved CBF in the intrathoracic pressure regulation group compared with the control group as measured by a Bowman Perfusion Monitor® probe. The current study suggests that the same improvement in CerPP could similarly result in an actual improvement in blood flow in humans, although CBF was not measured in this study.

One advantage of aIPR compared to other treatments for brain injury is that it is noninvasive and does not require drug or fluid injections. We hypothesize that the CerPP and ICP remained favorable after discontinuation of therapy due to the faciliation of the venous drainage caused by the use of the device. This is one of the key features of this technology as no other intervention other than head positioning and head elevation improves venous drainage. The relationship between respiratory mechanics and hemodynamics was first recognized by Moreno *et al.* showing the effect of an inhalation on inferior vena cava flow [13]. The physiological responses to the noninvasive devices developed to regulate changes in ITP and to provide greater circulatory support have been summarized by Convertino *et al.* [14]. Reduced ITP lowers right atrial pressure, which in turn pulls more venous blood back into the thorax. We speculate that this venous blood also comes from the brain resulting in increased venous drainage of all fluids in the brain including the CSF, resulting in the lowering of ICP.

The hemodynamic effect of utilization of negative pressure during the decompression phase of resuscitation was initially demonstrated during cardiopulmonary resuscitation [15]. The new aIPR approach presented in this paper has been used in animals and patients in cardiac arrest and noncardiac arrest shock states. During cardiopulmonary resuscitation, aIPR by increasing organ blood return to the heart and perfusion can improve ETCO_2_, a marker of circulation during resuscitation, and return of spontaneous circulation [16]. After cardiac arrest, aIPR has been studied for ICP management in which aIPR demonstrated that reduction of ITP to subatmospheric levels resulted in an instantaneous and sustained reduction in ICP in spontaneously breathing and apneic animals [17]. It is important to note that this effect was most pronounced in hypovolemic animals [18]. Furthermore, it has been demonstrated that part of the hemodynamic effect is due to an increase in cardiac output [19]. We speculate that the effect on ICP is due to the lowering of right atrial pressure resulting in improved venous drainage.

In the different studies that have been performed with aIPR, no significant gas exchange abnormalities have been observed, although all studies have been performed on animals and/or patients with uncompromised lungs: PaO_2_/FiO_2_ (P/F) ratio > 250. Comparative studies focused on histopathology of lung sections in traditionally ventilated pigs compared to pigs subjected to aIPR for 24 hours showed no differences in the level of atelectasis and edema, or lung compliance (unpublished data). These studies also determined that shunting does occur during the highest level of aIPR (− 12 cmH_2_O), but was limited to approximately 15% compared to traditional positive pressure mechanical ventilation with 5 cmH_2_O PEEP.

This human translational study has several limitations. The cost of the commercial device has not been established yet and may be a future limitation. We recognize that low enrollment in this study may have been due to the exclusion criteria that limited the recruitment of a significant number of patients with brain injury. In particular, due to the nature of this therapy and its desired effect on ITP there was a necessity to exclude potential participants who presented with cardiac or pulmonary injury impacting ITP and/or cardiac function and participants with respiratory disease, as many patients with brain injury also present with some kind of respiratory injury or can develop lung injury after being placed on a ventilator. In addition, treatment was limited to only 2 hours and duration of compromised cerebral flow is typically substantially longer in head injury. Finally, the exact role of this approach whether it be utilized as a first-line defense, in combination with osmotic agents to reduce ICP, or whether it be utilized as a salvage therapy, has not been determined yet and further study will be necessary.

## Conclusions

These data support the notion that aIPR, in this limited evaluation of patients with brain injury, can successfully augment CerPP by lowering ICP and increasing MAP in brain injured patients. The measured effects were immediate on administration of the therapy and persisted to some degree after the therapy was terminated.

## References

[1] Thurman DJ, Alverson C, Dunn KA, Guerrero J, Sniezek JE. Traumatic brain injury in the United States: a public health perspective. J Head Trauma Rehabil. 1999;14:602–15. 10.1097/00001199-199912000-00009 10671706

[2] Sosin DM, Sniezek JE, Waxweiler RJ. Trends in death associated with traumatic brain injury, 1979 through 1992. Success and failure. JAMA. 1995;273:1778–80. 7769773

[3] Chesnut RM, Marshall LF, Klauber MR, Blunt BA, Baldwin N, Eisenberg HM, Jane JA, Marmarou A, Foulkes MA. The role of secondary brain injury in determining outcome from severe head injury. J Trauma. 1993;34:216–22. 10.1097/00005373-199302000-00006 8459458

[4] Max W, Rice DP, MacKenzie EJ. The lifetime cost of injury. Inquiry. 1990;27:332–43. 2148306

[5] Changaris DG, McGraw CP, Richardson JD, Garretson HD, Arpin EJ, Shields CB. Correlation of cerebral perfusion pressure and Glasgow coma scale to outcome. J Trauma. 1987;27:1007–13. 10.1097/00005373-198709000-00009 3656462

[6] Murray LS, Teasdale GM, Murray GD, Miller DJ, Pickard JD, Shaw MD. Head injuries in four British neurosurgical centres. Br J Neurosurg. 1999;13:564–9. 10.1080/02688699943060 10715724

[7] The Brain Trauma Foundation. The American Association of Neurological Surgeons. The Joint Section on Neurotrauma and Critical Care. Hypotension. J Neurotrauma. 2000;17:591–5. 10.1089/neu.2000.17.591 10937905

[8] Metzger A, Rees J, Kwon Y, Matsuura T, McKnite S, Lurie KG. Intrathoracic pressure regulation improves cerebral perfusion and cerebral blood flow in a porcine model of brain injury. Shock. 2015;44(Suppl 1):96–102. 10.1097/SHK.0000000000000314 25692250

[9] Metzger A, Rees J, Segal N, McKnite S, Matsuura T, Convertino VA, Gerhardt RT, Lurie KG. "Fluidless" resuscitation with permissive hypotension via impedance threshold device therapy compared with normal saline resuscitation in a porcine model of severe hemorrhage. J Trauma Acute Care Surg. 2013;75:S203–9. 10.1097/TA.0b013e318299d5d0 23883909

[10] Metzger A, Matsuura T, McKnite S, Lurie K. The intrathoracic pressure regulator improves cerebral perfusion pressures and carotid blood flow in a porcine model of traumatic brain injury. Circulation. 2008;118:664.

[11] Metzger A, Matsuura T, McKnite S, Marino B, Nadkarni V, Lurie K, Yannopoulos D. The intrathoracic pressure regulator improves hemodynamics and 24-hour survival in a pediatric porcine model of severe hemorrhagic shock. Circulation. 2010;122:Abstract 10.

[12] Kiehna EN, Huffmyer JL, Thiele RH, Scalzo DC, Nemergut EC. Use of the intrathoracic pressure regulator to lower intracranial pressure in patients with altered intracranial elastance: a pilot study. J Neurosurg. 2013;119:756–9. 10.3171/2013.4.JNS122489 23706051

[13] Moreno AH, Burchell AR, Van der Woude R, Burke JH. Respiratory regulation of splanchnic and systemic venous return. Am J Phys. 1967;213:455–65. 10.1152/ajplegacy.1967.213.2.455 6036333

[14] Convertino VA, Ryan KL, Rickards CA, Glorsky SL, Idris AH, Yannopoulos D, Metzger A, Lurie KG. Optimizing the respiratory pump: harnessing inspiratory resistance to treat systemic hypotension. Respir Care. 2011;56:846–57. 10.4187/respcare.01018 PMC6607101 21333089

[15] Lurie KG, Voelckel WG, Zielinski T, McKnite S, Lindstrom P, Peterson C, Wenzel V, Lindner KH, Samniah N, Benditt D. Improving standard cardiopulmonary resuscitation with an inspiratory impedance threshold valve in a porcine model of cardiac arrest. Anesth Analg. 2001;93:649–55. 10.1097/00000539-200109000-00024 11524335

[16] Segal N, Parquette B, Ziehr J, Yannopoulos D, Lindstrom D. Intrathoracic pressure regulation during cardiopulmonary resuscitation: a feasibility case-series. Resuscitation. 2013;84:450–3. 10.1016/j.resuscitation.2012.07.036 22902465

[17] Yannopoulos D, McKnite SH, Metzger A, Lurie KG. Intrathoracic pressure regulation for intracranial pressure management in normovolemic and hypovolemic pigs. Crit Care Med. 2006;34:S495–500. 10.1097/01.CCM.0000246082.10422.7E 17114984

[18] Yannopoulos D, Metzger A, McKnite S, Nadkarni V, Aufderheide TP, Idris A, Dries D, Benditt DG, Lurie KG. Intrathoracic pressure regulation improves vital organ perfusion pressures in normovolemic and hypovolemic pigs. Resuscitation. 2006;70:445–53. 10.1016/j.resuscitation.2006.02.005 16901611

[19] Huffmyer JL, Groves DS, Desouza DG, Littlewood KE, Thiele RH, Nemergut EC. The effect of the intrathoracic pressure regulator on hemodynamics and cardiac output. Shock. 2011;35:114–6. 10.1097/SHK.0b013e3181f7d92b 20926988

